# Effects of Substituting *Tenebrio molitor* and *Elodea nuttallii* as Feed on Growth, Flesh Quality and Intestinal Microbiota of Red Swamp Crayfish (*Procambarus clarkii*)

**DOI:** 10.3390/foods13142292

**Published:** 2024-07-20

**Authors:** Muyan Li, Weiwei Lv, Yifan Zhao, Weiwei Huang, Quan Yuan, Hang Yang, Aimin Wang, Wenzong Zhou, Mingyou Li

**Affiliations:** 1 Key Laboratory of Integrated Rice-Fish Farming, Ministry of Agriculture and Rural Affairs, Shanghai Ocean University, Shanghai 201306, China; limuyan0720@163.com (M.L.); 17854232836@163.com (Y.Z.); 2 Eco-Environmental Protection Research Institute, Shanghai Academy of Agricultural Sciences, Shanghai 201403, China; wwlv@saas.sh.cn (W.L.); hwwswx@163.com (W.H.); quanyuan2016@126.com (Q.Y.); yhangqu2024@163.com (H.Y.); 3 Key Laboratory of Integrated Rice-Fish Farming, Ministry of Agriculture and Rural Affairs, Shanghai Academy of Agricultural Sciences, Shanghai 201403, China; 4 College of Marine and Biology Engineering, Yancheng Institute of Technology, Yancheng 224051, China; blueseawam@ycit.cn

**Keywords:** feed substitution, *Procambarus clarkii*, growth, nutrition, intestinal microbiota

## Abstract

This study aimed to evaluate the impact of substituting a portion of feed with *Tenebrio molitor* (TM) and *Elodea nuttallii* (EN) on crayfish culture. A total of 270 crayfish (5.1 ± 0.4 g) were fed three different diet combinations (A: 100% feed; B: 80% feed + 10% TM + 10% EN; C: 75% feed + 15% TM + 10% EN) for 12 weeks. The findings demonstrated that group C had an important beneficial impact on the growth performance of crayfish. This was evidenced by a rise in digestive enzyme activity (trypsin, lipase, and cellulase) in the intestinal and hepatopancreas, as well as an upregulation in the expression of growth-related genes (*ghsr*, *igfbp7*, *mhc*, *mlc1*, *mef2*, and *pax7*) in the muscle. Furthermore, the assessment of the flesh quality of crayfish muscle in group C was conducted. The findings indicated a significant increase (*p* < 0.05) in the energy value (moisture, crude protein, and crude lipid) within the muscle. The levels of delicious amino acids (Glu, Ala, Ser, Gly, and Tyr) and polyunsaturated fatty acids (ARA, DHA) were enhanced, resulting in an improved nutritional profile and flavor of the muscle while maintaining the Σn-3/Σn-6 ratio. The remodeling of the intestinal microbiota (abundance of *Proteobacteria* and ratio of *Firmicutes*/*Bacteroidota* bacteria) also revealed improved growth performance. Additional research is necessary to ascertain whether excessive use of TM or EN feed substitution can have negative effects on crayfish culture.

## 1. Introduction

Wild fisheries supplies have experienced a decline in recent years as a result of global climate issues and excessive fishing. Nevertheless, the issue of food security is becoming increasingly urgent on a worldwide scale due to population growth [1]. Given that aquatic products serve as a significant protein source, aquaculture is widely seen as a viable approach to attaining food security [2,3,4].

Fishmeal (FM) is widely acknowledged as a major protein source in feed and serves an essential function in the area of aquaculture. Nevertheless, the scarcity of FM sources and the growing need for them have resulted in a persistent escalation in costs [5]. In order to attain sustainability in aquaculture, it is imperative to significantly decrease reliance on FM as a feed source [6]. The suitability of insect proteins as alternatives to FM in aquafeeds has emerged as a prominent area of research in the current academic landscape. A growing amount of research indicates that the yellow mealworm (*Tenebrio molitor*, TM) is the second most extensively researched insect in aquaculture, following the black soldier fly (*Hermetia illucens*) [7]. Under dry conditions, TM exhibits elevated levels of crude protein (47–60%) and lipids (31–43%), while displaying comparatively low ash content (<5%), this characteristic renders them potentially valuable sources of high-quality proteins [8]. Substituting a portion of the FM can have beneficial impacts on the diet habits of numerous aquatic animals, such as turbot (*Scophthalmus maximus*) [9], largemouth bass (*Micropterus salmoides*) [10], mirror carp (*Cyprinus carpio* var. specularis) [11], blackspot sea bream (*Pagellus bogaraveo*) [12], rainbow trout (*Oncorhynchus mykiss*) [13,14], and sea bass (*Dicentrarchus labrax* L.) [15]. Studies on crustaceans have also discovered similar positive effects, such as pacific white shrimp (*Litopenaeus vannamei*) [16,17,18] and giant river prawn (*Macrobrachium rosenbergii*) [19].

Macrophytes possess the potential to transform aquaculture by serving as another alternative to FM, thereby addressing the increasing worldwide need for protein [20,21]. Crustaceans are generally omnivorous aquatic organisms, and stable isotope methods used to analyze crustacean food sources indicate that macrophytes account for most of the contribution [22,23]. Elodea (*Elodea nuttallii*, EN) is commonly utilized in crustacean aquaculture because of its abundant nutrients, strong tillering ability, and capacity to thrive in cold conditions [24]. Currently, there is a lack of studies in crayfish on different combinations of the two FM alternatives.

The quality and safety of fish meat are of paramount importance to the consumer, and therefore, the selection of fishmeal substitutes should not only be limited to the effect on growth performance but, more importantly, to the evaluation of muscle quality parameters. Flesh quality is a multifaceted characteristic, with healthiness (nutritional quality) being a significant factor in the consumer’s perception of flesh quality [25]. The nutrient content of food is primarily governed by the composition of its proteins, fatty acids, and amino acids. Meats that include a significant number of proteins, essential amino acids, and a high percentage of polyunsaturated fatty acids are considered to have good nutritional quality [26].

Recent research has demonstrated that the intestinal flora significantly affects the gastrointestinal health of aquatic species [27,28,29]. The intestinal microbiota produces digestive enzymes that facilitate the growth and development of aquatic organisms [30]. Research on crayfish has demonstrated that dietary sources can reshape the composition of intestinal microbes in a short period of time, and specific probiotics can enhance their growth performance [31]. In order to better understand the effects of feed replacement on microbial interactions in crayfish, it is necessary to include microbiome analyses.

Red swamp crayfish (*Procambarus clarkii*, *P. clarkii*) have gained significant importance in China as an irreplaceable and economically important aquatic product due to their distinct flavor and rich nutritional content, making them highly desirable among consumers. The aquaculture output of crayfish is steadily growing each year, with a significant market demand [32]. In 2022, aquaculture production reached a substantial 2,890,684 tons. The primary goals of this study were (i) to examine the impact of using natural baits as a substitute for fishmeal on the growth performance, meat quality, and gut flora of crayfish; (ii) to assess the viability of various proportions of TM and EN alternatives; and (iii) to offer novel perspectives for future crayfish aquaculture in order to discover alternative protein sources for the purpose of decreasing feed expenses.

## 2. Materials and Methods

### 2.1. Experimental Diets

The basic feed is a commercial pellet feed (Hubei Zhaoliang Biotechnology Co., Ltd., Xiantao, Hubei, China), the pellet was finely ground to powder using a grinder and then filtered using an 80-mesh sieve. TM and EN were obtained from self-raising in the Zhuanghang Comprehensive Experiment Station of the Shanghai Academy of Agricultural Sciences and freeze-dried in a freeze-dryer until a constant weight was reached. The freeze-dried TM and EN were finely ground to powder using a grinder and then filtered using an 80-mesh sieve.

This study was divided into three distinct feeding groups through the mixing of varying proportions of the powdered feeds: Group A is 100% feed and serves as the control group; Group B is 80% feed + 10% TM + 10% EM; and Group C is 75% feed + 15% TM + 10% EN. The TM and EN ratios were chosen with reference to our previous pretests of different TM and EN ratios for the short-term feeding of crayfish (Supplementary Figure S1). All proportionally mixed powders were extruded through a single-screw extruder (SLP-45, Chinese Fishery Machinery and Instrument Research Institute) at 85 ± 5 °C to sinking pellets (2 mm), and all pellets were air-dried, sealed, and stored at 4 °C, following a study by [33].

### 2.2. Experimental Animals

Crayfish were provided by Shanghai Xiangsheng Aquaculture Farming Co-operative Society Ltd. (Shanghai, China). Before the experiment, crayfish were temporarily kept in a concrete tank measuring 3.0 m × 5.0 m × 0.5 m for a period of 7 days to acclimate to the experimental environment. The tank was located in the recirculating water culture greenhouse at the Zhuanghang Comprehensive Experiment Station of the Shanghai Academy of Agricultural Sciences. During this period, all crayfish were provided with equal quantities of pellet feed.

### 2.3. Experimental Design and Management

A total of 270 crayfish (9.4 ± 0.2 g) in optimal condition were chosen and allocated at random to nine concrete tanks (1.0 m × 1.0 m × 0.4 m) for the purpose of recirculating water culture. Each experimental group (A, B, and C) comprised three replicates, with each replicate consisting of 30 crayfish. Shelter consisting of a sufficient amount of PVC pipes and crayfish nests at the bottom of the tank. The crayfish are fed at 17:00 daily, during which the recirculating water circulation is temporarily halted. During the feeding process, we provide a quantity of food equivalent to 4–5% of the body weight; each tank is provided with an equal quantity of food until all crayfish in each tank are fully satisfied, and any remaining food is promptly removed.

The feeding experiment took place in the Zhuanghang Comprehensive Experiment Station for a duration of 12 weeks, spanning from May to July. The photoperiod was maintained at 14L:10D, and the daily death rate of crayfish is accurately documented. The tank’s water quality is regularly assessed five times a day using a portable water quality analyzer (model HQ40D, HACH, Loveland, CO, USA) to measure water temperature, pH levels, and dissolved oxygen (DO). NH_4_^+^-N, NO_2_N, and chemical oxygen demand (COD) levels were quantified using chemical methods, and Table 1 shows the parameters of each indicator.

### 2.4. Sample Collection

After 12 weeks, all crayfish were anesthetized on ice after 24 h of fasting. Subsequently, the weight and quantity of crayfish in each tank were calculated, and vernier calipers were utilized to obtain a precise measurement of the carapace’s length. The crayfish were then dissected, and the muscle and hepatopancreas were carefully removed and measured for weight.

Three crayfish were selected at random from each tank. The foregut, hepatopancreas, and parts of muscle were gathered and stored at −80 °C for subsequent enzyme activities and quantitative polymerase chain reaction. Another part of the muscle was stored at −40 °C for subsequent flesh quality determination. 

Two crayfish were selected at random from each tank. The hindgut was gathered and stored at −80 °C for intestinal microbiota determination.

### 2.5. Growth Performance Indicators

The growth performance indices in this experiment were determined using the following formulae:

IBW: Initial body weight (g).
FBW: Final body weight (g).
SR: Survival rate (%) = 100 × final number of crayfish/initial number of crayfish.
WGR: Weight gain rate (%) = 100 × (FBW − IBW)/IBW.
SGR: Specific growth rate (%/d) = 100 × (ln FBW − ln IBW)/84 d.
HSI: Hepatosomatic index (%) = 100 × hepatopancreas weight/FBW.
MY: Meat yield (%) = 100 × muscle weight/FBW.
CF: Condition factor (g/cm^3^) = FBW/(carapace’s length)^3^.

### 2.6. Sample Pretreatment and Determination Methods

#### 2.6.1. Digestive Enzyme Activities

The intestinal and hepatopancreas were rinsed with ice-cold phosphate-buffered saline (PBS) and then sliced into minute fragments. Tissue homogenates were acquired using recently prepared ice-cold saline (1:10 *w*/*v*). Subsequently, the homogenate was subjected to centrifugation at a speed of 4000 rpm for 10 min at 4 °C, the supernatant was then gathered and kept for measurement. The activities of digestive enzymes (amylase, trypsin, lipase, and cellulase) were quantified using commercially available kits (Nanjing Jiancheng Bioengineering Institute Co., Ltd., Nanjing, China).

#### 2.6.2. Muscle Routine Biochemical Composition

The moisture content was determined by subjecting the samples to freeze-drying in a freeze-dryer until a stable weight was attained. The Kjeldahl method was used to determine the crude protein content. The total fat content was determined by extracting the crude fat from the samples using a low-boiling-point organic solvent (ether or petroleum ether; Shanghai Reagent Corp., Shanghai, China) in a process called Soxhlet extraction, the difference in weight between the samples and the residue was used to calculate the crude fat content. The ash content was determined by subjecting the samples to high temperatures (550 °C) in a muffle furnace until a constant weight was obtained.

#### 2.6.3. Muscle Amino Acid Content and Composition

Weighed precisely 100 mg (accurate to 1 mg) of freeze-dried muscle powder. Then, samples were hydrolyzed with a 6 mol/L HCl solution at 110 °C for 24 h. The hydrolysate was then filtered through a 0.22 µm membrane and analyzed using an automatic amino acid analyzer (model L-8900, Hitachi, Tokyo, Japan).

#### 2.6.4. Muscle Fatty Acid Content and Composition

We precisely weighed 500 mg (accurate to 10 mg) of the homogeneous muscle sample into a 10 mL stoppered test tube and added 5 mL of chloroform–methanol solution (2:1, *v*/*v*) for 60 min. The mixture was filtered and evaporated to obtain the total lipid. We added 1 mL of 1 mol/L potassium–hydroxide–methanol solution at 60 °C for 30 min, then methylated it with a 12.5% sulfuric acid–methanol reagent. The upper solution was then separated by adding 2 mL of distilled water and 1 mL of n-hexane. The organic phase was washed with a saturated sodium chloride solution three times, and then subjected to centrifugation at a speed of 4000 rpm for 10 min; the supernatant was taken for determination by GC-MS (model 7980B, Agilent, Santa Clara, CA, USA), following a study by [34].

#### 2.6.5. Muscle RNA Extraction and Quantitative Polymerase Chain Reaction (qPCR)

A Trizol reagent (Sigma, Burlington, VT, USA) was utilized to extract the total RNA from the muscle tissue. The reverse transcription was conducted in accordance with the guidelines provided by the cDNA synthesis kit (TaKaRa, Kusatsu, Japan). The gene sequence was obtained from GenBank, and qPCR primers (Table 2) were synthesized by Sangon Biotech Co., Ltd. (Shanghai, China).

The Roche LightCycler^®^480 II Real-Time System (Roche, Basel, Switzerland) was used to perform qPCR, and the SYBR Green PCR Master Mix Kit (TaKaRa, Kusatsu, Japan) was utilized for this purpose. Each reaction system consisted of 10 µL of SYBR mix, 6.4 µL of double distilled water (ddH_2_O), 0.8 µL of forward primer, 0.8 µL of reverse primer, and 2 µL of cDNA as the template. A two-step PCR reaction procedure was used: pre-denaturation at 95 °C for 30 s, 40 cycles of denaturation at 95 °C for 5 s, and annealing at 60 °C for 20 s. Following each qPCR experiment, a melting curve analysis was conducted on the products to verify their specificity. The relative abundance of target gene mRNA was calculated by R = 2^−ΔΔCt^, *β-actin* was utilized as a calibration standard to standardize the expression of the target genes, and each sample was replicated three times.

#### 2.6.6. Intestinal DNA Extraction and Microbiota Composition Analysis

The QIAamp DNA Stool Mini Kit (QIAGEN, Hilden, Germany) was utilized to extract the genomic DNA from the intestine, and then the concentration of DNA was detected by NanoDrop 2000 (Thermo Fisher Scientific, Waltham, MA, USA).

Sequencing was performed by the Illumina NovaSeq 6000 platform (Illumina Inc., San Diego, CA, USA; OE Biotech Co., Ltd., Shanghai, China). Compositional analysis of intestinal microbiota such as α-diversity, β-diversity, and abundance was carried out by the OE Cloud Platform.

### 2.7. Statistical Analysis

The data were computed using Microsoft Excel and analyzed using SPSS 22.0 software. The groups were assessed for significant differences using one-way analysis of variance (ANOVA), followed by Tukey’s test. The data were presented as means ± SD (standard deviation), and statistical significance was determined as a *p*-value < 0.05.

## 3. Results

### 3.1. Growth Performance

Table 3 showed a significant rise (*p* < 0.05) in the FBW, WGR, SGR, HSI, and MY of crayfish in group C when compared with groups A and B. There was no statistically significant difference (*p* > 0.05) in the SR and CF of crayfish when comparing the three different diet combinations.

### 3.2. Digestive Enzyme Activities

**Figure 1:**
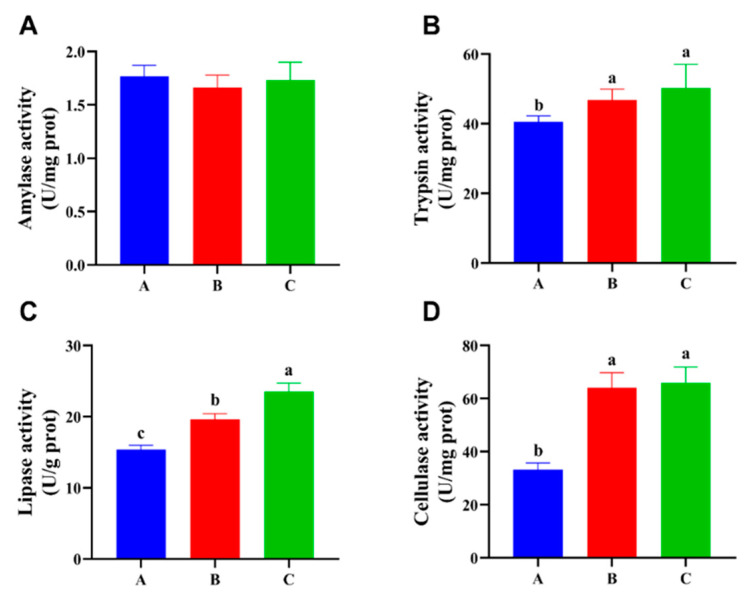
Effects of three different diet combinations on intestinal digestive enzyme activity levels of *P. clarkii after* 12 weeks (n = 9). Different lowercase letters indicate significant differences among the groups (*p* < 0.05). (**A**–**D**) Amylase, trypsin, lipase, and cellulase activity levels in the intestinal.

**Figure 2:**
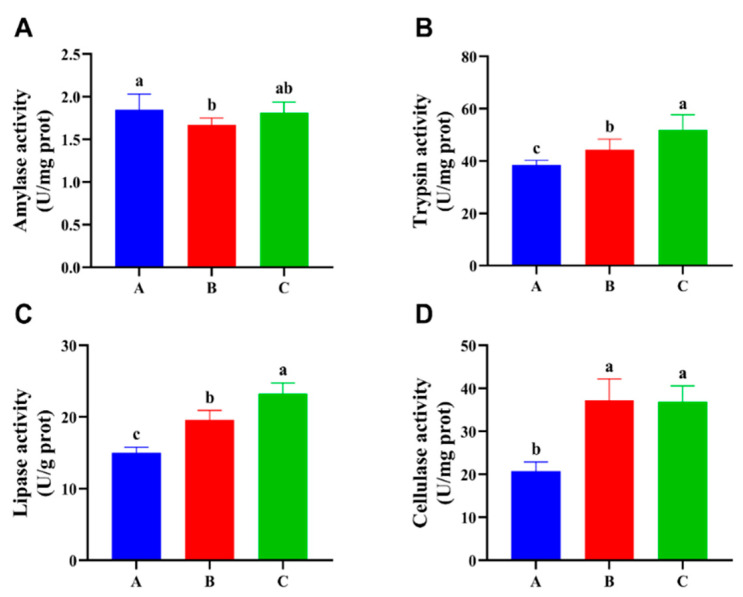
Effects of three different diet combinations on hepatopancreas digestive enzyme activity levels of *P. clarkii* after 12 weeks (n = 9). Different lowercase letters indicate significant differences among the groups (*p* < 0.05). (**A**–**D**) Amylase, trypsin, lipase, and cellulase activity levels in the hepatopancreas.

The levels of nutritive absorption were investigated by testing the activity of digestive enzymes, as shown in Figure 1 and Figure 2. The results show no statistically significant difference (*p* > 0.05) in amylase activity across the three different diet combinations in the intestine, but trypsin, lipase, and cellulase activity were significantly increased (*p* < 0.05) in group C compared with group A. The activity of trypsin, lipase, and cellulase in the hepatopancreas of group C was similar to that in the intestinal, and all exhibited a significant increase (*p* < 0.05) compared with group A. However, group A exhibited significantly higher amylase activity (*p* < 0.05) compared with the other two groups.

### 3.3. Muscle Proximate Composition

According to the data presented in Table 4, the muscle moisture content of crayfish in groups B and C was substantially higher than that of group A (*p* < 0.05). Additionally, the muscle crude protein and crude lipid content in group C was substantially higher (*p* < 0.05) compared with groups A and B. Nevertheless, there was no significant difference in the ash content of the muscles among the three groups (*p* > 0.05).

### 3.4. Muscle Amino Acid Composition

Table 5 reveals that the muscles of crayfish in all experimental groups had a total of 15 amino acids, consisting of 7 essential amino acids, 8 nonessential amino acids, and 5 taste-active amino acids. The total amino acids (TAA) content exhibited a substantial increase (*p* < 0.05) in response to the higher quantity of yellow mealworms consumed across all three diet combinations. The muscle’s essential amino acids (EAA) content and the ratio of EAA/TAA in groups A and B were significantly higher (*p* < 0.05) than in group C. The content of nonessential amino acids (NEAA) in the muscles of groups A and B were significantly lower (*p* < 0.05) compared with group C. The content of delicious amino acids (DAA) in groups B and C was substantially higher (*p* < 0.05) compared with group A.

### 3.5. Muscle Fatty Acid Composition

Table 6 displays the presence of 21 fatty acids in the crayfish muscle across all experimental groups, and these include 8 total saturated fatty acids, 4 monounsaturated fatty acids, and 9 polyunsaturated fatty acids. Out of the three different diet combinations, the muscle SFA level in group A was significantly higher (*p* < 0.05), whereas the PUFA, n-3 PUFA, and n-6 PUFA content were significantly lower (*p* < 0.05) compared with groups B and C. The primary fatty acids found in crayfish muscle include palmitic acid (C16:0), oleic acid (C18:1n-9), linoleic acid (C18:2n-6), and arachidonic acid (C20:4n-6).

### 3.6. Muscle Growth and Development-Related Genes Expression

**Figure 3:**
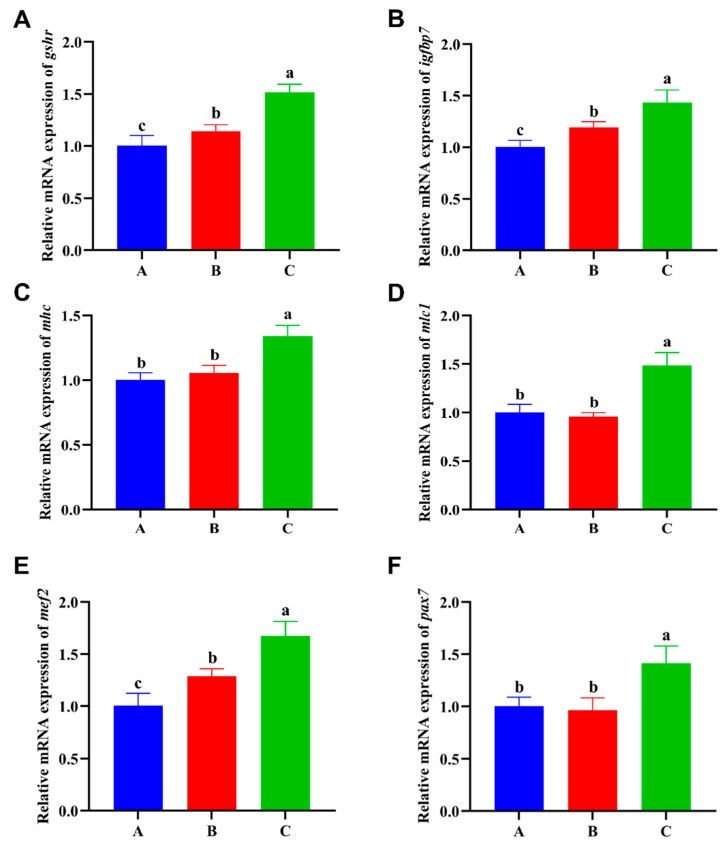
Effects of three different diet combinations on muscle mRNA expression levels of *P. clarkii* after 12 weeks (n = 9). Different lowercase letters indicate significant differences among the groups (*p* < 0.05). (**A**–**F**) *ghsr*, *igfbp7*, *mhc*, *mlc1*, *mef2*, and *pax7* mRNA expression levels in the muscle.

The mRNA expression levels of genes associated with muscle growth and development were evaluated to study performance under three different diet combinations (Figure 3). Compared with Group A, the mRNA expression of all genes in Group C was significantly upregulated (*p* < 0.05).

### 3.7. Intestinal Microbiota Diversity and Composition

**Figure 4:**
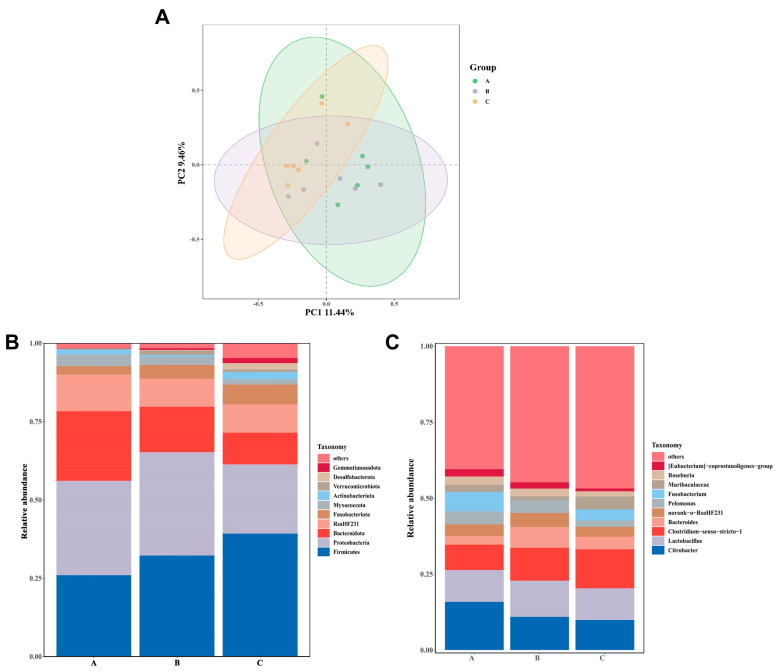
Effects of three different diet combinations on the composition of the intestinal microbiota in *P. clarkii* after 12 weeks (n = 6). (**A**) Principal coordinates analysis (PCoA) results. (**B**) Average relative abundances at the phylum level. (**C**) Average relative abundances at the genus level.

Table 7 shows the alpha diversity of crayfish intestinal flora for the three dietary combinations. There was good coverage above 99% in all three groups, guaranteeing the dependability of the diversity outcomes. In the observed species, the Shannon, Simpson, and Chao1 indices exhibited a statistically significant rise in groups B and C as compared with the control group (*p* < 0.05), reflecting a higher level of richness and diversity in the intestinal flora. Figure 4A displays the PCoA results based on the OUT levels, calculated using the Bray–Curtis distance.

Figure 4B,C displays the top 10 phyla and genera in each group and their corresponding abundance data. At the phylum level, the dominant bacteria (average relative abundance > 10%) in group A are *Proteobacteria* (30.07%), *Firmicutes* (26.01%), *Bacteroidota* (22.22%), and *RsaHF231* (11.79%); in group B *Proteobacteria* (33.02%), *Firmicutes* (32.25%), and *Bacteroidota* (14.47%); and in group C *Firmicutes* (39.27%), *Proteobacteria* (22.10%), and *Bacteroidota* (10.09%). At the genus level, the dominant bacteria (average relative abundance > 5%) in group A are *Citrobacter* (15.88%), *Lactobacillus* (10.48%), *Clostridium_sensu_stricto_1* (8.34%), and *Fusobacterium* (6.40%); in group B *Lactobacillus* (11.91%), *Citrobacter* (10.93%), *Clostridium_sensu_stricto_1* (10.82%), and *Bacteroides* (6.85%); and in group C *Clostridium_sensu_stricto_1* (12.80%), *Lactobacillus* (10.46%), and *Citrobacter* (9.90%).

## 4. Discussion

Recently, there has been a growing interest in finding alternatives to fishmeal in animal feed for aquaculture. This study aims to investigate the viability of partially replacing TM and EN for feed. Regrettably, the current study did not include trials with a higher proportion of TM substitution groups due to site constraints. However, the current findings are still encouraging.

Group C exhibited significant enhancements in FBW, WGR, SGR, HSI, and MY compared with groups A and B, as observed through growth performance analysis. It is important to point out that an increase in body weight does not necessarily indicate an increase in meat yield but is sometimes associated with an increased weight of internal organs, and the results of group C show an increase in the proportion of meat yield as well. Previous research has observed that this phenomenon mostly arises from the enhanced efficiency of nutrient utilization in fish. The intestinal microbiota of TM has chitin and chitosan materials, which are believed to possess immunostimulatory properties and enhance the growth of fish [35,36]. Conversely, an excessive level of TM substitution might have a detrimental impact on the growth performance of fish. The probable explanation for this phenomenon is that as the concentration of TM rises, it accumulates as indigestible carbohydrates, and higher levels of chitin may impede the normal working of the digestive system, negatively affecting growth performance [37,38]. Further study is required to ascertain the optimal rate of TM substitution in crayfish diets.

We detected statistically significant alterations in the growth performance of crayfish, with the intestinal and hepatopancreas identified as the primary organs responsible for the digestion and absorption of nutrients. Assessing the enzymatic activity of the digestive enzymes in these two organs aided in clarifying a specific feature of the enhanced growth performance. The digestive enzymes of crustaceans are essential to the regulation of the growth and molting cycles [39]. The results of our investigation indicate that groups B and C showed varying increases in both lipase and trypsin activity when compared with the control group. The observed effect may be related to the substitution of a portion of the feed with TM, akin to the observations made in tilapia (*Oreochromis niloticus*) [40] and rainbow trout (*Oncorhynchus mykiss*) [41]. The significant increase in cellulase activity, on the other hand, is more likely to be attributable to the partial replacement of feed by EN. The supplementation of exogenous cellulase is believed to boost fish growth by enhancing the activity of digestive enzymes and improving intestinal flora [42,43]. This implies that the substitution of a portion of the feed with TM and EN enhances the nutrient absorption capacity of crayfish, providing a more intuitive explanation for the enhanced growth performance observed in crayfish. The aquaculture business is seeing significant growth, bringing an increased supply of aquatic products. Consequently, there is a growing emphasis on research pertaining to flesh quality in order to supply more high-quality aquatic products. The proximate composition of fish flesh encompasses the quantification of moisture, protein, fat, and ash content. Examining these constituents enables us to evaluate the fish’s energy worth [44]. This study’s findings indicate a significant increase in the levels of moisture, crude protein, and crude lipid in group C. The increase in levels of crude protein and crude fat in flesh may be attributed to the high protein and fat content of TM itself, which enhances the body’s ability to utilize and convert these nutrients. The rise in their moisture content can be attributed to the superior growth, development, and weight gain performance of groups B and C compared with group A, resulting in more frequent molting. Typically, recently shed crayfish have a greater amount of water in their bodies due to their more pliable structure. Alternatively, the inclusion of EN components in the meal could potentially result in an augmentation of muscle water content, which needs to be further explored.

In order to further investigate the stimulatory impact of TM heel EN on crayfish growth, the expression of various genes associated with muscle growth and development was analyzed. Ghrelin activates the ghrelin–GHSR axis and its effect on AMPK to stimulate functions such as feeding and growth hormone release [45]. The expression of *ghsr* was shown to be significantly elevated in Nile tilapia (*Oreochromis niloticus*) [46], exhibiting high growth performance, and Chinese mitten crab (*Eriocheir sinensis*) [47] displaying precociousness, as determined through transcriptome analysis. *igfbp7* has a role in cell differentiation, proliferation, and growth, either through an IGF-dependent or -independent model [48]. The involvement of *igfbp7* in the development of germ cells and gonads was observed in studies conducted on Chinese sole tongue (*Cynoglossus semilaevis*) [49]. Therefore, it may be inferred that *igfbp7* plays a pivotal role in the mechanisms of growth and development. The hexamer structure of myosin, which consists of two heavy chains and two sets of nonidentical light chains, performs structural and kinetic functions during muscle growth and movement [50,51]. The impact of *mhc* and *mlc*, which are the primary constituents of thick muscle filaments, on the process of thick muscle filaments synthesis and muscle growth has been demonstrated [52,53,54]. The family of myocyte enhancer factor 2 (*mef2*) has four distinct subtypes (*mef2a*, *mef2b*, *mef2c*, *mef2d*) that exert significant influence over genetic programs, including cell proliferation and differentiation [55]. Paired box 7 (*pax7*) is crucial in the process of tissue and organ creation during development [37]. The role of *pax7* in muscle development has been demonstrated in several aquatic organisms, such as Japanese flounder (*Paralichthys olivaceus*) [56], zebrafish (*Danio rerio*) [57], and Japanese pufferfish (*Takifugu rubripes*) [58]. The current research unveiled a significant upregulation in the expression of all genes within group C. This implies that the inclusion of TM and EN can enhance the activation of these genes, hence promoting the growth of crayfish. However, the precise regulatory mechanisms and pathways involved require additional investigation.

The assessment of protein quality depends mainly on the analysis of amino acid composition and content [59,60]. One crucial aspect that affects the flavor of flesh is the content of DAA. Ser, Gly, and Ala present a sweet taste, while Glu presents an umami taste. The amino acids Gly, Lys, Phe, and Thr have the ability to undergo a Maillard reaction with glucose, resulting in the production of various aromas [61]. Concurrently, research conducted on crucian carp (*Carassius auratus*) [62] and Senegalese sole (*Solea senegalensis*) [63] has demonstrated that elevated levels of Glu and Ala enhance the quality of their flesh. Within group C, the levels of all the aforementioned amino acids exhibited a significant elevation, with the exception of a minor decline in Phe. In studies of other FM substitutes, replacing FM with high proportions of *Clostridium autoethanogenum* protein [64] and Antarctic krill meal [65] reduced DAA levels in muscles. Likewise, substituting FM with a high level of TM leads to a comparable outcome, and it is highly probable that an ideal level of TM substitution also exists in crayfish, although this remains to be investigated in greater detail.

The fatty acid content and composition in muscle tissue are of utmost importance for the nutritional value of fish and greatly influence its flavor [66]. Research findings indicate that increasing the intake of UFA and reducing the intake of SFA is an effective strategy for preventing cardiometabolic disease [67,68]. Both groups B and C exhibited a significant decrease in SFA content and a significant increase in UFA content as compared with the control group. Research conducted on freshwater crayfish (*Astacus leptodactylus*) revealed that including dietary n-3 PUFAs resulted in enhanced nutritional quality of the muscle and hepatopancreas, while having no impact on the levels of cholesterol and vitamins in the muscle [69]. Furthermore, it is generally accepted that having higher proportions of Σn-3/Σn-6 fatty acids is thought to be beneficial, especially EPA and DHA, as they play a role in controlling inflammation and safeguarding against cardiovascular disease [70]. In contrast, the role of ARA in the development of aquatic organisms is intricate, the precise mechanism by which it regulates growth remains unknown. Nevertheless, an increasing amount of research indicates that optimizing dietary ARA levels has beneficial effects on growth, immunological function, and antioxidant capacity [71,72,73], and our results support this view. Collectively, these findings indicate that the substitution of TM and EN for certain diets resulted in a positive impact on the quality of meat. However, the specific mechanism behind this improvement requires additional investigation.

The intestinal microbiota has a crucial role in influencing the nutritional status and growth of aquatic animals [74]. Modifications in food composition can result in alterations in the composition of the intestinal microbiota. Our study findings demonstrate that the dominant phyla in the intestinal microbiota of crayfish are *Proteobacteria*, *Firmicutes*, and *Bacteroidota*, which aligns with earlier research [75]. The phylum *Proteobacteria* is commonly found in the intestinal flora of crustaceans and plays a crucial role in several biochemical cycles, such as carbon, nitrogen, and sulfur cycling [76,77]. The excessive abundance of the *Proteobacteria* phylum is believed to have a detrimental impact on crustaceans, as it hinders their growth performance and raises their risk of disease [78,79]. *Firmicutes* have been shown to utilize dietary fiber via fermentation and also engage with the intestinal mucosa, thus playing a role in maintaining homeostasis in the body [80,81]. Recent research indicates that there is growing evidence linking high *Firmicutes*/*Bacteroidota* ratios to obesity and the deposition of fat in animals [82,83]. At the phylum level, the abundance of the *Firmicutes* phylum showed an increasing trend from group A to group C, while that of the *Bacteroidota* phylum showed a decreasing trend. This result is consistent with our conclusion. *Citrobacter*, classified under the phylum *Proteobacteria*, is commonly recognized as an opportunistic pathogen in aquatic animals, causing a variety of diseases and inhibiting their growth performance [84,85]. Opportunistic pathogens often pose no threat as they are benign constituents of the regular microbial population. Nevertheless, an imbalance in their population might result in negative effects [86]. As a potential probiotic, increased *Clostridium* abundance has been shown to improve growth performance and immunity [87]. Therefore, we hypothesized that replacing a portion of the feed with TM and EN would not only enhance the variety and amount of intestinal microorganisms but also result in some restructuring of the bacterial flora, improve the function of cooperative digestive enzymes, enhance the digestion and absorption of nutrients, stimulate body growth, decrease the likelihood of opportunistic pathogen infection, and enhance the body’s immune response.

## 5. Conclusions

In general, the substitution of feed with a combination of 15% TM and 10% EN proved to be effective at enhancing the growth performance of crayfish. This was supported by an increase in the activity of digestive enzymes and an upregulation of growth-related genes in the muscle. Furthermore, there was a substantial rise in the energy value within the muscle. The levels of DAAs and PUFAs were enhanced, resulting in an improved nutritional profile and flavor of the muscle while maintaining the Σn-3/Σn-6 ratio. Furthermore, the intestinal microbiota underwent remodeling, providing an additional explanation for the enhanced growth performance.

## Figures and Tables

**Table 1:** Water quality indicator parameters.

Parameters	Numeric Range
Temperature (°C)	20–25
pH	7.52–8.41
DO (mg/L)	6.0–7.0
NH_4_^+^-N (mg/L)	0.042–0.053
NO_2_N (mg/L)	0.031–0.039
COD (mg/L)	12.0–14.7

**Table 2:** Primer sequence for qPCR.

Genes	Primers (5′–3′)	Accession No.
*ghsr*	F: 5′ GCCTTCTTGAGGGTGTTC 3′ R: 5′ GTTGGTGCCGTAGTTGAG 3′	XM_045728526.1
*igfbp7*	F: 5′ GGGACAAATGCGACAGGA 3′ R: 5′ AAGAAGTGGGTGACGAGGAA 3′	XM_045757043.1
*mhc*	F: 5′ TTCGGTATGGATCTTATGG 3′ R: 5′ GAACACGGGAGACTTGC 3′	XM_045726227.1
*mlc1*	F: 5′ CCATCTTCGCTCAGGTT 3′ R: 5′ GTACATCATAGTGCCATTCTC 3′	XM_045743365.1
*mef2*	F: 5′ TCACCGCCCGTTAGGAT 3′ R: 5′ GCCCAGGATGGTTGGAAGA 3′	XM_045740701.1
*pax7*	F: 5′ ATGACGGTAACAGGAGCG 3′ R: 5′ ATGGCGGAGACTGAGGG 3′	XM_045750481.1
*β-actin*	F: 5′ AGGTTGCTGCCCTGGTT 3′ R: 5′ CACGCTTGCTCTGTGCC 3′	XM_045725824.1

Note: *ghsr*: growth hormone secretagogue receptor; *igfbp7*: insulin-like growth-factor-binding protein 7; *mhc*: myosin heavy chain; *mlc1*: myosin light chain 1; *mef2*: myocyte-specific enhancer factor 2; *pax7*: paired box 7.

**Table 3:** Effects of three different diet combinations on growth performance of *P. clarkii* after 12 weeks.

Parameters	A	B	C	*p*-Value
IBW (g)	9.03 ± 0.19	9.08 ± 0.21	9.02 ± 0.22	0.439
FBW (g)	37.25 ± 3.47 ^b^	36.22 ± 2.17 ^b^	44.85 ± 1.68 ^a^	<0.001
SR (%)	88.67 ± 0.58	90.67 ± 1.53	89.67 ± 2.08	0.343
WGR (%)	312.77 ± 39.36 ^b^	299.01 ± 25.88 ^b^	397.59 ± 24.30 ^a^	<0.001
SGR (%/d)	1.68 ± 0.11 ^b^	1.64 ± 0.08 ^b^	1.91 ± 0.06 ^a^	<0.001
HSI (%)	6.88 ± 0.63 ^b^	5.32 ± 0.87 ^c^	8.12 ± 1.01 ^a^	<0.001
MY (%)	10.38 ± 0.57 ^b^	10.10 ± 0.39 ^b^	11.11 ± 0.78 ^a^	<0.001
CF (g/cm^3^)	0.043 ± 0.002	0.044 ± 0.004	0.045 ± 0.003	0.073

Note: Values are presented as means ± SD (standard deviation). Superscript letters within the same row represent statistically significant differences (*p* < 0.05).

**Table 4:** Muscle proximate composition of three different diet combinations of *P. clarkii* after 12 weeks (n = 9).

Parameters	A	B	C	*p*-Value
Moisture (%)	63.57 ± 1.17 ^b^	73.20 ± 1.64 ^a^	74.50 ± 2.86 ^a^	<0.001
Crude protein (%)	16.56 ± 0.96 ^b^	17.17 ± 0.79 ^b^	21.31 ± 1.17 ^a^	<0.001
Crude lipid (%)	2.12 ± 0.14 ^b^	2.11 ± 0.46 ^b^	2.71 ± 0.45 ^a^	0.003
Ash (%)	2.34 ± 0.15	2.33 ± 0.34	2.12 ± 0.30	0.170

Note: Values are presented as means ± SD (standard deviation). Superscript letters within the same row represent statistically significant differences (*p* < 0.05).

**Table 5:** Muscle amino acid composition (g/100g) of three different diet combinations of *P. clarkii* after 12 weeks (n = 9).

Parameters	A	B	C
Threonine	0.77 ± 0.03 ^b^	0.99 ± 0.04 ^a^	0.96 ± 0.77 ^a^
Valine	0.53 ± 0.04 ^b^	0.42 ± 0.02 ^c^	0.63 ± 0.03 ^a^
Isoleucine	0.33 ± 0.02	0.33 ± 0.02	0.31 ± 0.01
Leucine	0.80 ± 0.05 ^a^	0.33 ± 0.03 ^b^	0.30 ± 0.01 ^b^
Phenylalanine *	0.42 ± 0.02 ^a^	0.40 ± 0.02 ^a^	0.26 ± 0.01 ^b^
Lysine	3.30 ± 0.18 ^a^	3.07 ± 0.18 ^b^	1.47 ± 0.08 ^c^
Methionine	0.28 ± 0.03 ^a^	0.27 ± 0.03 ^a^	0.22 ± 0.01 ^b^
**ΣEAA**	6.42 ± 0.24 ^a^	5.81 ± 0.21 ^b^	4.15 ± 0.09 ^c^
Serine	0.97 ± 0.04 ^c^	1.30 ± 0.03 ^b^	1.68 ± 0.11 ^a^
Glutamate *	1.37 ± 0.12 ^c^	1.61 ± 0.15 ^b^	2.47 ± 0.10 ^a^
Glycine *	1.86 ± 0.08 ^b^	2.59 ± 0.12 ^a^	1.95 ± 0.05 ^b^
Alanine *	1.63 ± 0.05 ^b^	1.98 ± 0.08 ^a^	1.92 ± 0.12 ^a^
Histidine	1.78 ± 0.09 ^c^	2.34 ± 0.07 ^b^	3.68 ± 0.15 ^a^
Arginine	1.57 ± 0.11 ^a^	1.37 ± 0.12 ^b^	1.18 ± 0.07 ^c^
Tyrosine *	0.30 ± 0.04 ^a,b^	0.34 ± 0.03 ^a^	0.29 ± 0.04 ^b^
Proline	0.65 ± 0.06 ^b^	0.56 ± 0.04 ^b^	3.62 ± 0.24 ^a^
**ΣNEAA**	10.12 ± 0.13 ^c^	12.08 ± 0.35 ^b^	16.79 ± 0.16 ^a^
**ΣDAA**	5.58 ± 0.16 ^b^	6.91 ± 0.23 ^a^	6.89 ± 0.17 ^a^
**ΣTAA**	16.55 ± 0.25 ^c^	17.89 ± 0.49 ^b^	20.94 ± 0.23 ^a^
**ΣEAA/ΣTAA (%)**	38.81 ± 0.99 ^a^	32.47 ± 0.72 ^b^	19.81 ± 0.24 ^c^

Note: Values are presented as means ± SD (standard deviation). Superscript letters within the same row represent statistically significant differences (*p* < 0.05). ΣEAA: total essential amino acids; ΣNEAA: total nonessential amino acids; ΣDAA: delicious amino acids; ΣTAA: total amino acids. The parameter with the mark “*” in the upper-right corner indicates DAA.

**Table 6:** Muscle fatty acid composition (% of total fatty acids) of three different diet combinations of *P. clarkii* after 12 weeks (n = 9).

Parameters	A	B	C
C12:0	0.08 ± 0.01	0.08 ± 0.00	0.09 ± 0.01
C14:0	0.35 ± 0.01 ^c^	0.57 ± 0.05 ^b^	0.77 ± 0.15 ^a^
C15:0	0.12 ± 0.02 ^b^	0.10 ± 0.01 ^b^	0.17 ± 0.03 ^a^
C16:0	14.47 ± 0.83 ^a^	12.97 ± 0.56 ^b^	15.02 ± 0.51 ^a^
C17:0	0.87 ± 0.08 ^a^	0.41 ± 0.04 ^b^	0.48 ± 0.04 ^b^
C18:0	13.51 ± 0.32 ^a^	11.01 ± 0.49 ^b^	7.71 ± 0.19 ^c^
C19:0	0.57 ± 0.01 ^b^	0.62 ± 0.04 ^a^	0.42 ± 0.01 ^c^
C20:0	0.29 ± 0.01	0.29 ± 0.03	0.27 ± 0.02
**ΣSFA**	30.25 ± 0.92 ^a^	26.04 ± 0.65 ^b^	24.93 ± 0.45 ^c^
C16:1n-7	7.85 ± 0.41 ^a^	5.79 ± 0.18 ^b^	4.68 ± 0.26 ^c^
C17:1n-7	0.68 ± 0.09 ^b^	0.83 ± 0.06 ^a^	0.82 ± 0.07 ^a^
C18:1n-9	16.65 ± 0.98 ^b^	15.33 ± 0.38 ^c^	23.56 ± 0.61 ^a^
C20:1n-9	1.00 ± 0.04 ^b^	1.27 ± 0.06 ^a^	0.96 ± 0.02 ^b^
**ΣMUFA**	26.18 ± 1.11 ^b^	23.23 ± 0.50 ^c^	30.02 ± 0.70 ^a^
C18:2n-6	11.76 ± 1.07 ^b^	14.93 ± 0.89 ^a^	10.81 ± 0.9 ^b^
C18:3n-3	2.14 ± 0.09 ^c^	2.94 ± 0.13 ^b^	3.19 ± 0.21 ^a^
C20:2n-6	3.31 ± 0.12 ^c^	4.43 ± 0.07 ^a^	3.59 ± 0.11 ^b^
C20:3n-6	3.37 ± 0.09 ^a^	3.33 ± 0.12 ^a^	3.02 ± 0.23 ^b^
C20:4n-6(ARA)	8.28 ± 0.31 ^c^	8.88 ± 0.41 ^b^	10.57 ± 0.32 ^a^
C20:5n-3(EPA)	7.50 ± 0.29 ^b^	8.43 ± 0.18 ^a^	5.25 ± 0.15 ^c^
C22:2n-6	1.12 ± 0.08 ^b^	1.34 ± 0.11 ^a^	0.94 ± 0.05 ^c^
C22:4n-6	1.02 ± 0.06 ^b^	1.37 ± 0.08 ^a^	0.79 ± 0.08 ^c^
C22:6n-3(DHA)	5.07 ± 0.04 ^b^	5.08 ± 0.11 ^b^	6.89 ± 0.10 ^a^
**ΣPUFA**	43.57 ± 1.22 ^c^	50.73 ± 1.02 ^a^	45.05 ± 0.84 ^b^
**Σn-3 PUFA**	14.72 ± 0.30 ^c^	16.45 ± 0.25 ^a^	15.33 ± 0.37 ^b^
**Σn-6 PUFA**	28.86 ± 1.02 ^b^	34.27 ± 1.03 ^a^	29.72 ± 0.76 ^b^
**Σn-3/Σn-6(%)**	51.04 ± 1.48 ^a^	48.05 ± 1.72 ^b^	51.62 ± 1.85 ^a^

Note: Values are presented as means ± SD (standard deviation). Superscript letters within the same row represent statistically significant differences (*p* < 0.05). ΣSFA: total saturated fatty acids; ΣMUFA: total monounsaturated fatty acids; ΣPUFA: total polyunsaturated fatty acids; Σn-3 PUFA: total n-3 polyunsaturated fatty acids; Σn-6 PUFA: total n-6 polyunsaturated fatty acids.

**Table 7:** Bacterial alpha diversity of three different diet combinations of *P. clarkii* after 12 weeks (n = 6).

Parameters	A	B	C	*p*-Value
Goods coverage (%)	99.76 ± 0.06 ^b^	99.88 ± 0.09 ^a^	99.90 ± 0.06 ^a^	0.011
Observed species	488.25 ± 64.87 ^b^	635.72 ± 62.41 ^a^	697.22 ± 74.82 ^a^	<0.001
Shannon	4.27 ± 0.51 ^b^	5.71 ± 0.70 ^a^	6.29 ± 0.64 ^a^	<0.001
Simpson	0.88 ± 0.02 ^b^	0.92 ± 0.02 ^a^	0.93 ± 0.02 ^a^	0.002
Chao1	485.60 ± 64.94 ^b^	633.53 ± 63.52 ^a^	694.35 ± 73.76 ^a^	<0.001

Note: Values are presented as means ± SD (standard deviation). Superscript letters within the same row represent statistically significant differences (*p* < 0.05).

## Data Availability

The original contributions presented in the study are included in the article/Supplementary Material, further inquiries can be directed to the corresponding author.

## Supplementary Materials

The following supporting information can be downloaded at https://www.mdpi.com/article/10.3390/foods13142292/s1. Figure S1: Specific growth rates of crayfish under alternative commercial feed conditions with different percentages of TM (A) and EN (B) after 4 weeks.
