# Supplementary material for: Effects of Substituting Tenebrio molitor and Elodea nuttallii as Feed on Growth, Flesh Quality and Intestinal Microbiota of Red Swamp Crayfish (Procambarus clarkii)
Source: Foods. 2024 Jul 20;13(14):2292. doi: 10.3390/foods13142292 (PMC11275352; doi:10.3390/foods13142292)
Supplement: Supplementary file 1 [file foods-13-02292-s001.zip › foods-3078713-supplementary.pdf]

Based on our previous pre-experiment, nine trials with different feed combinations were set up, Group A with 70% commercial feed + 30% yellow mealworm (*Tenebrio molitor*, TM), Group B with 80% commercial feed + 20% TM, Group C with 85% commercial feed + 15% TM, Group D with 90% commercial feed + 10% TM, Group E with 70% commercial feed + 30% Elodea (*Elodea nuttallii*, EN), Group F with 80% commercial feed + 20% EN, Group G was 85% commercial feed + 15% EN, Group H was 90% commercial feed + 10% EN, and Group I was 100% commercial feed (control group).

The culture was conducted using 90 L plastic tanks. A total of 270 crayfish were evenly distributed among 27 plastic tanks, with every 3 tanks forming a group. Continuous aeration was provided throughout the culture period, and PVC pipes and shrimp tanks were used as shelters, taking advantage of natural light and water temperature. The experiment utilized ambient light and controlled water temperature. Feeding occurred every day at 17:00. Feces and erbium residues were removed by suction using a siphon tube. The number of deceased shrimp was tallied on a daily basis, and the weight and quantity of shrimp in each tank were measured and recorded after 4 weeks. 1/3 of the water body was replaced every day after feeding.

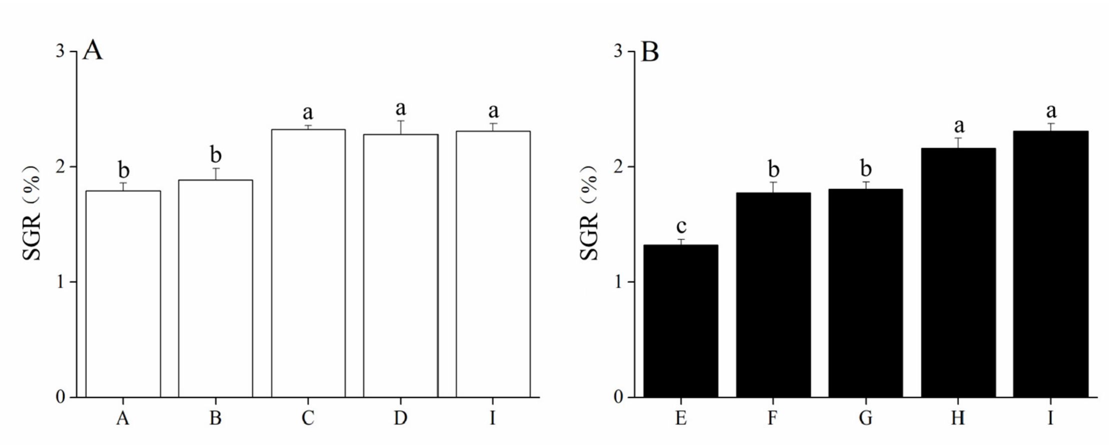

Figure S1. Specific growth rates of crayfish under alternative commercial feed conditions with different percentages of TM (**A**) and EN (**B**) after 4 weeks.

Specific growth rate of crayfish was significantly ($P < 0.05$) higher ($P < 0.05$) than that of 20-30% replacement group using TM as a replacement for 10-15% commercial feed and it was not significantly ($P > 0.05$) different from the commercial-only feed. The specific growth rate of crayfish was significantly higher ($P < 0.05$) than that of the 15-20% replacement group and not significantly different ($P > 0.05$) from that of the 100% commercial feed group with the use of EN as a replacement for 10% of the commercial feed, whereas the specific growth rate of crayfish in the 30% EN replacement group was significantly lower ($P < 0.05$) than that of the 10-20% replacement group.

Therefore, we chose the two feed combinations from the formal experiment: 80%feed + 10%TM + 10%EN; 75%feed + 15%TM + 10%EN.
